# On the Relation between the Development of Working Memory Updating and Working Memory Capacity in Preschoolers

**DOI:** 10.3390/jintelligence10010005

**Published:** 2022-01-21

**Authors:** Sabrina Panesi, Alessia Bandettini, Laura Traverso, Sergio Morra

**Affiliations:** 1 National Research Council of Italy, Institute for Educational Technology, 16149 Genoa, Italy; 2 Department of Education, University of Genoa, 16128 Genoa, Italy; alessia.bandettini@edu.unige.it (A.B.); laura.traverso@unige.it (L.T.)

**Keywords:** updating, working memory, M capacity, executive function, preschoolers

## Abstract

This study aims at investigating the relationship between working memory updating and working memory capacity in preschool children. A sample of 176 preschoolers (36–74 months) was administered a working memory updating task (Magic House) along with three working memory capacity tests that specifically measure their core attentional component (M capacity, as defined in the theory of constructive operators): Backward Word Span, Mr. Cucumber, and Direction Following Task. Correlational analyses and cross-classification prediction analyses were performed. Updating and capacity were significantly correlated, although the correlations were not high when age was partialled out. Capacity increased with age, and mediated the relation between age and updating. More importantly, cross-classification prediction analysis revealed that high updating scores with low M capacity, and low updating scores with relatively high M capacity, are possible events; the only combination ruled out was a low updating score with precocious development of M capacity. These facts demonstrate that updating skills in preschoolers depends on M capacity but does not coincide with it. Therefore, in cognitive developmental theories, the constructs of working memory updating and capacity should be distinguished, and on practical grounds, different tests should be used to measure them.

## 1. Introduction

This article deals with the relation between working memory capacity and updating. In line with current research and theory (e.g., Cowan 2017) we define working memory as a system that can hold a limited amount of information in a heightened state of availability for use in ongoing information processing. The capacity of this system, i.e., the amount of information that can be held simultaneously, is assumed to depend on limited, domain-free attentional resources that are used to activate the task-relevant schemes or representations (Barrouillet et al. 2004; Cowan 2005; Engle et al. 1999; Pascual-Leone and Johnson 2021; Ruchkin et al. 2003; Vergauwe et al. 2012). In the literature, working memory capacity is measured with different tasks, depending on both practical considerations (e.g., the age of the participants in an experiment) and the assumptions of the specific theoretical framework within which a study is carried out; nevertheless, the results are often comparable across studies that operationalize working memory capacity with different tasks. Most theories assert that children’s working memory capacity increases with age (e.g., Barrouillet et al. 2009; Cowan 2016; Pascual-Leone and Johnson 2005).^1^ We define executive functions, following Miyake et al. (2000), as a set of circumscribed, lower-level functions that are general-purpose mechanisms involved in the control of cognition by modulating the operation of other cognitive subprocesses. Updating is one of the basic executive functions identified by Miyake and colleagues. It was first defined by Morris and Jones (1990) as the act of modifying the current status of a representation or schema in working memory to accommodate new input. Miyake et al. (2000, p. 57) define it as the monitoring and updating of working memory representations, i.e., “monitoring and coding incoming information for relevance to the task at hand and then appropriately revising the items held in working memory by replacing old, no longer relevant information with newer, more relevant information”.

Updating is an important cognitive function, which is involved in different activities. For example, when we read a novel, we need to maintain new relevant representations and inhibit old, no-longer-relevant representations, in order to understand the text (e.g., Iglesias-Sarmiento et al. 2015; Palladino et al. 2001). Likewise, during a mental arithmetic operation, updating allows for the storage of intermediate results in working memory that can be manipulated to find the final result, while discarding the information that is no longer needed. Updating information from the external environment is crucial for maintaining correspondence between the external world and internal representation. Moreover, working memory representations can be updated from the internal world: this is essential for high-level cognition, such as arithmetic calculation, language comprehension, and reasoning. Finally, updating information with a sub-goal or a task-relevant context is crucial for goal-directed behavior, in which the required actions cannot rely on overlearned long-term routines (Rac-Lubashevsky and Kessler 2016). This explains the importance of updating skill in adaptation.

Furthermore, if intelligence is essential to adaptation, it is not surprising that the relation between intelligence and executive functions has been examined in the literature. It has been reported that updating is critical to fluid intelligence in both adults and children. Friedman et al. (2006), in a study on a large sample of adolescents, found that updating correlated highly with both fluid and crystallized intelligence, whereas inhibition and shifting—i.e., the other two executive functions specified in Miyake et al.’s (2000) model—were unrelated to fluid and only weakly related to crystallized intelligence. Belacchi et al. (2010) found a high correlation, in a sample of children aged 4 to 11, between fluid intelligence (measured with Raven’s colored matrices) and a measure of updating, even though the updating task used verbal materials. A meta-analysis (Au et al. 2015) of studies on adults indicates that training on a working memory updating task, the *n*-back, has a significant transfer effect on fluid intelligence test performance. This result was challenged by other meta-analytic studies controlling for moderating variables and potential artifacts (Melby-Lervåg et al. 2016; Shipstead et al. 2012).^2^ Other recent meta-analyses with further controls (Au et al. 2016; Soveri et al. 2017), however, confirmed the existence of a significant training effect. Overall, the current balance seems to indicate a small but significant effect of n-back training on fluid intelligence. Therefore, both correlational and training studies suggest that updating contributes to individual differences in fluid intelligence.

An important open question about cognitive functioning is the relationship between working memory capacity and updating. In fact, in literature there are contrasting claims about the overlapping or differentiation of these two constructs. In adult research, updating and working memory are regarded as two correlated but distinct constructs. Miyake et al. (2000) identified a three-component model in which they distinguished three different though related executive functions (inhibition, shifting and updating) and suggested that all of them rely on working memory. Based on this model, several researchers consider updating as an executive function in the service of working memory. Himi et al. (2021) investigated the factor structure of executive function (inhibition, shifting, and updating) and other cognitive constructs, such as working memory capacity, relational integration, and divided attention in adults. They found a five-factor structure which included the first factor predominately loading shifting tasks, the second factor loading two updating tasks, the third predominately representing working memory capacity, the fourth consisting of relational integration and antisaccade tasks and, finally, the last factor consisting of divided attention and stop signal tasks. Therefore, updating and working memory capacity turned out to be separated latent variables, albeit correlated (*r* = .57). On the contrary, another study indicated a high correlation (*r* = .96) between updating assessed with *n*-back tasks and working memory capacity tapped by a set of content-heterogeneous tasks (Schmiedek et al. 2009), suggesting that the two constructs coincide. However, most experimental and correlational studies suggest a relatively weak relationship between working memory capacity and updating (see Redick and Lindsey 2013 for a meta-analysis showing that n-back and complex span tasks cannot be used interchangeably; complex span tasks are often used to measure working memory capacity, especially in the frameworks of Engle’s and Barrouillet’s models). Moreover, practice on the *n*-back task does not improve performance on working memory capacity measures (e.g., Jaeggi et al. 2008; Redick et al. 2013). Additionally, Kane et al. (2007) reported nonsignificant to weak correlations between working memory capacity and updating tasks.

However, in the developmental literature, working memory capacity and updating are often considered as synonyms. Garon et al. (2008), for example, published a review about executive functioning in preschoolers, identifying inhibition, shifting, and working memory as executive functions. Likewise, Diamond (2013) considered inhibition, working memory, and cognitive flexibility as executive functions developing in young children. In those highly influential articles, working memory is considered an executive function, which replaces updating in Miyake et al.’s (2000) model.

On the other hand, there is another research tendency which argues that there is a substantial difference between the concepts of updating and working memory already in preschoolers (e.g., Morra et al. 2018). This assertion is consistent not only with the theoretical definitions of updating and capacity but also with empirical findings. Panesi and Morra (2017) designed an updating task called Magic House, in which children must remember, on each trial, the last two animals that entered sequentially into a house (i.e., a child version of a running span task), and found that this task correlated with capacity measures but correlated even higher with measures of inhibition (Panesi and Morra 2020). In a similar way, Traverso et al. (2015) used an adapted version of the Keep Track task, an updating task that, in five-year-olds, turned out to correlate primarily with inhibition measures and, to a lesser extent, with a working memory capacity measure. These results suggest that updating tasks are related to both working memory capacity and inhibition and, perhaps even more strongly, to inhibition than to working memory capacity. Therefore, they already support the distinction between measures of updating and working memory capacity in young children.

The confusion in the developmental literature between updating and capacity could be partly justified because few updating tasks for young children are available. However, this confusion in terminology also causes a confusion in the selection of measures: studies that consider updating and working memory capacity as synonyms use tasks relating to these constructs indiscriminately. On the other hand, researchers who consider these functions as differentiated skills employ different tasks to measure them.

Clarity in terminology, and in the use of validly interpretable measures, presupposes clarity of theoretical models. Our distinction between updating and capacity in children (as well as in adults) is consistent with a developmental model by Im-Bolter et al. (2006) which, in turn, is based on the Theory of Constructive Operators (e.g., Pascual-Leone and Johnson 2005). Pascual-Leone (1970, 1987) introduced the concept of M capacity, i.e., a limited domain-general attentional resource for activating task-relevant schemes that grows by maturation during childhood. This construct is similar to other recent working memory models highlighting the role of attentional resources in keeping relevant information active. Cowan (2005), for example, defines a limited “focus of attention” as the main determinant of working memory capacity; Engle et al. (1999) attribute a similar role to “controlled attention”; and Barrouillet et al. (2004) to “attentional resources” that re-activate relevant information, preventing its decay. There is a rising consensus that the growth of a limited attentional resource that explains working memory capacity is essentially due to maturation (Cowan 2016; Pascual-Leone and Johnson 2021), although Pascual-Leone’s theory is more detailed in describing its developmental trend. In particular, typical three-year-olds would have attentional resources sufficient to activate only one unit of information (i.e., a mental representation or a Piagetian scheme of symbolic nature); the capacity of two units is typically achieved at age five (Pascual-Leone 1970) or four and a half (Case 1985); and the capacity of three units at age seven (Pascual-Leone 1970) or six and a half (Case 1985). In the following, we use the term “M capacity” when we refer specifically to Pascual-Leone’s developmental model, or to measures conceived in the framework of that model; in other cases, we use the more generic term “working memory capacity”.

According to Im-Bolter et al. (2006), a modified version of Miyake et al.’s model can be integrated with the Theory of Constructive Operators, in the form of a two-layer, four-component model. In particular, they assume that there is a first, more basic layer including two general resources (inhibition and M capacity), and a second layer that includes executive functions proper, such as shifting and updating, both of which draw upon the basic resources of inhibition and M capacity. Therefore, this model provides clear theoretical grounds for the distinction between M capacity and updating.

Panesi and Morra (2020) found that a distinction between M capacity and inhibition measures emerged in preschoolers, and measures of shifting and updating were related more closely to inhibition than they were related to M capacity. These findings are consistent with Im-Bolter et al. (2006), because they point to a distinction, already in preschoolers, between the two basic resources of capacity and inhibition; and they also suggest that updating does not coincide with capacity. However, further investigation is needed to clarify the developmental relation between M capacity and updating.

### The Present Study

This study tests the contrasting predictions derived from different theoretical models. A developmental model that considers working memory an executive function along with inhibition and shifting (Garon et al. 2008) implies that capacity and updating measures are closely related, because they both belong to working memory, and (unless because of error variance) children with low working memory capacity performing well on updating tasks, or vice versa, should not occur. In contrast, the Theory of Constructive Operators and, more specifically, the model of Im-Bolter et al. (2006) predict that capacity and updating measures can dissociate because they are distinct constructs; however, they should also be correlated because updating partly draws on M capacity resources. We speculate that full emergence of an updating skill could take place with a capacity of three units, which would enable a child to consider an item to be discarded, an item that is going to replace it, and a replacement operation. However, some initial form of updating may also be possible for young children by combining the activation and inhibition processes (which, in the Theory of Constructive Operators, are formalized as the M and I operators; see Pascual-Leone and Johnson 2021). Thus, even some children with a low M capacity might perform well on updating tasks, particularly if they have a well-developed inhibition which enables them to discard no-longer relevant information, or if the task context makes salient the distinction between relevant and irrelevant information. Nevertheless, the capacity to hold a larger amount of information should facilitate efficient updating; this implies the prediction that M capacity and updating are correlated, though distinguishable.

The aim of the present study was to investigate whether updating is linked to the core component of working memory (M capacity) and whether updating coincides with M capacity in preschoolers. To test this, we calculated correlations between an updating task and three M capacity tasks and conducted cross-classification prediction analysis between updating and M capacity. If there is a perfect overlap between working memory and updating then we should observe a high correlation between the updating task and the working memory capacity tasks, and a high updating performance in children with low M capacity should be an impossible event. On the contrary, if there is a distinction between working memory and updating then we should observe a lower correlation between the updating task and the working memory capacity tasks, and a high updating performance in children with low M capacity should be a possible event.

## 2. Materials and Methods

### 2.1. Participants

A total of 176 children, 36–74 months old (M = 56.36; SD = 9.81; 51% female), recruited in Italian pre-schools, took part in this study. All children were Italian preschoolers with typical development (i.e., no formal diagnosis of disability, language impairment, or behavior disorder). Recruitment took place in five public schools and one private, located in a northern region of Italy. This sample includes the 118 participants in the Panesi and Morra (2020) study, whose data we are reanalyzing here, and 58 new children in the same age range. It is a convenience sample: in the Panesi and Morra (2020) study, the participants were all of the children in the three preschools who met the requirements for participation; similarly, in the new study, we are testing all available children in other preschools, and the first 58 that we tested are included here. Parents provided informed consent for participation, and the study was conducted conforming to the ethical standards established by the Italian Psychology Association. The socio-economic status composition of all areas in which the preschools are located is very mixed, including both medium-low SES families and medium-high ones.

In the Panesi and Morra (2020) study, a Monte Carlo simulation with Muthén and Muthén’s (2002) procedure ensured that the sample size was sufficient for the structural equation modelling carried out there. This sample was almost 50% larger than in Panesi and Morra (2020). We are not aware of generally recognized criteria for sample size in cross-classification prediction analyses; however, based on our previous experience, we think that having found standard errors of Del < .15 in all such analyses (see the Results section below) ensures that the current sample was sufficiently large for our purpose.

### 2.2. Procedure

Each child was tested individually in a quiet room at the preschool by trained graduate students. As mentioned above, the sample includes children that were recruited in two larger studies (Panesi and Morra 2020, and another study currently in progress), in which each child was assessed with updating and three working memory capacity tasks, along with other tasks not considered in this article.^3^ At the end of the assessment procedure, each child received a small gift for their effort.

### 2.3. Measures

#### 2.3.1. Updating Task

The following task was used to assess updating.

*Magic House* (Panesi and Morra 2017). This is an updating task that assesses the constant monitoring and addition or deletion of working memory contents. On each item, the child was shown 3, 4, or 5 toy animals that were placed sequentially in a cardboard house (without knowing in advance the number of animals in a sequence, as in running span tests for older children and adults). Then, the child must recall the last two animals placed in the house, as the experimenter asked: “Which was the last animal who entered the house? ... And which other animal entered just before it?”. There were 9 items, each of which was scored 0, 1, or 2 points if the child recalled no, one, or two animals. Thus, the scores could range from 0 to 18. Panesi and Morra (2020) reported a Cronbach alpha of .724 for this task.

#### 2.3.2. Working Memory (M Capacity) Tasks

To assess M capacity, the following tasks were administered.

*Mr. Cucumber test* (Case 1985). The outline of an extraterrestrial figure, to which stickers in different colors had been attached, was displayed for 5 s per item. The child must then show, on an outline without colored stickers, the positions of the stickers. There were three items at each level from 1 to 8 stickers. The test was discontinued when a child failed all three items at a level. One point was given for each consecutive level on which a subject got at least two items correct, and one third of a point for each correct item above that level. Thus, the scores could range from 0 to 8. Panesi and Morra (2020) reported a Cronbach alpha of .653 for this task.

*Backward Word Span* (Morra 1994). The child was required to repeat lists of words in reverse order. There were three lists at each level from 2 to 7 words. The test was discontinued when a child failed all three lists at one level. One point was given for each consecutive level on which a subject got at least two items correct (including level 1 which cannot exist, because it is not possible to reverse the order of a single word, and therefore it was granted as correct by default), and one third of a point for each correct item above that level. Thus, the scores could range from 1 to 7. Panesi and Morra (2020) reported a Cronbach alpha of .789 for this task.

*Direction Following Task* (Pascual-Leone and Johnson 2005) This task requires children to follow oral directions of increasing complexity. We modified it for preschoolers, using tokens of different shapes (bicycle and boat), colors (white, yellow, green, blue, and red) and sizes (large and small) to be placed in boxes of different color and size. We only presented items in the form “place an X in a Y” (i.e., the three simplest levels of complexity of the test). There were three levels and five items at each level (e.g., level 1: “place a large boat in a red box”; level 2: “place a little yellow boat in a green box”; level 3: “place a large green boat in a little yellow box”). The scoring rules for the Italian version of the test (see Morra et al. 2013) were followed, adapting them for this shorter version. The score, based on a theory-guided task analysis, is assumed to represent a child’s M capacity (see Pascual-Leone and Johnson 2011). In particular, we gave 0 points if fewer than three responses at each level were correct; 1 point if at least three responses out of five were correct at the first level but fewer than three at the second and third levels; 2 points if at least four responses at the first level and at least three responses at the second level were correct, but fewer than three were correct in the third level, or if at least eight items were correct in total; 3 points if at least four responses at the first level, at least four responses at the second level, and at least three responses at the third level were correct. Most participants clearly performed according to one of the foregoing patterns. There were 16 children who performed very high (at least four responses out of five were correct at each level); because this short version without more complex levels does not enable one to discriminate whether such ceiling performance indicates a score of 3 or 4, we assigned them a score of 3.5. Finally, there were 13 children with an irregular pattern of performance; they were assigned a score of 1.5 or 2.5, i.e., the average between the score of the patterns that were closest to their actual performance. Thus, the scores could range from 0 to 3.5. Panesi and Morra (2020) reported a Cronbach alpha of .849 for this task.

## 3. Results

Descriptive statistics (mean, standard deviation, score range, skewness, and kurtosis) for the updating and working memory capacity tasks are shown in Table 1. Sufficiently large interindividual variability was recorded for most tasks, and no floor or ceiling effects were found.

Zero-order (Pearson) correlations between age, updating, and working memory capacity measures are displayed in Table 2.^4^ The Magic House was significantly correlated with all working memory capacity tasks. These correlations remained significant also with age partialled out, being *r*(173) = .20, *p* < .01 with the Backward Word Span, *r*(173) = .19, *p* < .02 with the Mr. Cucumber Test, and *r*(173) = .24, *p* < .01 with the Direction Following Task. Both zero-order and partial correlations are informative, because the former take into account both developmental and individual-difference variance, whereas the latter take into account only individual-difference variance. Note that the partial correlations, although significant, were relatively low—suggesting that updating is related to working memory capacity, but the two constructs do not coincide. Additionally, we calculated the correlation between the Magic House and the mean of the three working memory capacity tasks, which was *r*(174) = .52, *p* < .001, and remained significant when partialling out age, *r*(173) = .31, *p* < .001. It is useful to consider the mean of the three working memory capacity tasks, because averaging them yields a measure of capacity relatively free from content bias and other sources of impurity that characterize each single task. The observed pattern of correlations is consistent with the concept of a positive relation between working memory capacity and updating, at the levels of both developmental and individual differences.

It is also interesting to note that although scores on all tasks correlated significantly with age, this correlation was higher (in the range .53–.68) for M capacity tests than for the Magic House (.45); this difference was significant, *z* = 3.09, *p* < .01, according to Meng et al. (1992, Formula (6)). This indicates that the M capacity measures were more closely related to age than the Magic House was.

Moreover, the correlation between Magic House and age dropped below significance when the mean of the working memory capacity tasks was partialled out, *r*(173) = .11, *p* > .15. The converse, however, was not true; indeed, even when partialling out the Magic House scores, the correlation between working memory capacity and age remained high, *r*(173) = .67, *p* < .001. This suggests that the correlation between age and the Magic House was mediated by working memory capacity.

Subsequently, we performed the cross-classification prediction analysis (Hildebrand et al. 1977) that allows a researcher to develop a priori customized hypothesis that can test a theory of the relationship between the states of two variables. In particular, this analysis requires the researcher to hypothesize that a certain event is impossible, i.e., that the “true” frequency of cases in one or more “critical” cells of a cross-classification table is equal to 0. Of course, the observed value in the critical cells could actually be larger than 0 because of error variance. For the purpose of statistical inference, the index *Del* is defined, which indicates the extent to which the hypothesis that the critical cells are empty accounts for the data better than expected by chance (i.e., better than a hypothetical independence between rows and columns of the table). The computational formula for *Del* is (Exp–Obs)/Exp, where Exp and Obs are, respectively, the expected and observed frequencies in a set of critical cells. The maximum possible value of *Del* is 1, in case the observed frequencies in all critical cells are = 0. A confidence interval for *Del* is computed and, in case the confidence interval falls entirely in the positive range and includes the specific value of *Del* = 1, then one can accept the hypothesis that the critical event taken into consideration is impossible, i.e., does not really occur (and its occasional observation can be attributed merely to error variance).

If the constructs of working memory capacity and updating coincide (at least in children, as assumed by some researchers), then certain events should be impossible. In particular, children with a low capacity should not be able to attain high scores in an updating task. Moreover, children with average or above average capacity should not perform very low on an updating task.

To perform the analysis, performance on the Magic House was defined as low (0–9 points, i.e., ≤50% correct, which means no more than one animal per item on average), intermediate (10–13 points, i.e., between 51% and 75% correct), or high (14–18 points, i.e., >75% correct). We approximated, to the nearest unit, the average of the three working memory tasks. (Actually, the column for M capacity = 3 includes 33 children whose M capacity approximated to 3 and one child whose M capacity approximated to 4; because there was only one child with a capacity of four units, we did not make a specific column in the table, but we included this child in the “3” column). Table 3 presents the observed frequencies in the cross classification of Magic House performance by M capacity, along with the frequencies expected by chance. Here, and in the following sections, according to the rules for cross-classification prediction analysis, we call the frequencies expected under the assumption of independence between rows and columns “expected frequencies”.

The first critical event that we considered was the combination of a low M capacity (1 unit) with a high performance in the Magic House. There were nine children in this single cell, compared with an expected frequency of 23.9. This yielded *Del* = .62, with a standard error of *Del* S.E. = .12; *z* = 5.10, *p* < .001; the 99% C.I. of *Del* was (.31, .94). The significant *z* and the positive values of the whole C.I. indicate that this event was less frequent than chance; however, the fact that the C.I. did not include the value of 1 indicates that this event cannot be deemed “impossible”, i.e., that the nine observations falling in this cell could not be dismissed as mere error variance, and that the event under consideration does actually occur.

Another critical event that we considered was the combination of an average or above average M capacity (2 or 3 units) with a low performance in the Magic House. There were 10 + 1 = 11 children in these two cells, compared with a total expected frequency of 24.1. This yielded *Del* = .54, S.E. = .13, *z* = 4.08, *p* < .001, and the 99% C.I. was (.20, .89). Also in this case, it must be concluded that this event is less frequent than chance, but not impossible; the presence of children with average or above average capacity who perform low on the Magic House is a real event.

If we restrict our consideration to the combination of a high M capacity (3 units) with a low performance in the Magic House, there was one child in this single cell, compared with an expected frequency of 7.7. This yielded *Del* = .87, S.E. = .13; *z* = 6.72, *p* < .001, and a 99% C.I. of (.54, 1.20). In this case, the due conclusion is not only that this event is less frequent than chance, but that it also can be regarded as “impossible”, because the confidence interval includes the value of 1; in other words, one can accept the hypothesis that the real frequency of this event is zero, and that the one child falling into this cell can be accounted for by mere error variance. It is also interesting to note that the group of 34 children with a capacity of 3 units had a mean age of 65.6 months (s.d. = 5.7), i.e., they were five-year-olds with a larger capacity than the 2 units typically expected at their age. This group of five-year-olds, with precocious development of working memory capacity, performed high or intermediate, but not low, on our updating task.

## 4. Discussion

The relationship between updating and working memory is an important open issue. While they are considered separate constructs in adults, there is no accordance about their distinction in the developmental literature. Some authors, like Garon et al. (2008) and Diamond (2013), seem to consider that updating and working memory capacity are synonyms, identifying working memory as an executive function. On the other hand, another research tendency (e.g., Morra et al. 2018) argues that working memory capacity and updating are different constructs—consistent with more recent working memory models (e.g., Oberauer 2009) and, more specifically, with how working memory and executive functions are conceived in the theory of constructive operators (Im-Bolter et al. 2006; Pascual-Leone and Johnson 2021). However, in the literature, few studies have experimentally investigated the relationship between these two skills. For this reason, in the present study we aimed at investigating whether updating is related to the core, attentional component of working memory (called M capacity in Pascual-Leone’s theory) and whether updating coincides with M capacity in preschoolers.

Our findings revealed that the updating skill in preschoolers depends on M capacity, as indicated by a positive correlation that remained significant when partialling out age, but does not coincide with it. High updating scores with low M capacity, and low updating scores with at least average M capacity, are possible events which cannot be dismissed as error variance. These facts demonstrate that updating and M capacity do not coincide, although are correlated.

The pattern of correlations helps to clarify the nature of the relation between capacity and updating. First, working memory capacity was found to be highly correlated with age. Second, updating also correlated positively with age, but this correlation was actually mediated by capacity. This suggests that working memory capacity, which is closely related to age, develops essentially by maturation, and that updating, in turn, depends on capacity (but does not coincide with it). These findings are highly consistent with the theory of constructive operators (Im-Bolter et al. 2006; Pascual-Leone and Johnson 2021). This outcome is, instead, in contrast with the view that updating can be reduced to working memory in a general way, as assumed by Garon et al. (2008) and Diamond (2013).

Another interesting detail is the finding of one “impossible event” in the cross-classification prediction analyses, i.e., that children with a precocious (above average) development of working memory capacity did not perform low on our updating task. In a study on giftedness, Johnson et al. (2003) found that gifted schoolchildren had an M capacity larger than their peers by almost one unit. In a study on mathematical reasoning in schoolchildren, Agostino et al. (2010) found that updating mediated the relation between M capacity and mathematical problem solving. Although the present study was carried out with younger participants, we think that (by analogy with Johnson et al. 2003) we can regard our group of children with a capacity of 3 units as “gifted”; and these studies—including the present one—can be taken as further, indirect clues to the role of working memory capacity and updating ability in intellectual development. In light of our findings, future studies following up Belacchi et al.’s (2010) and Friedman et al.’s (2006) work on intelligence and updating should take in consideration the distinction between working memory capacity and updating and examine their respective (possibly intertwined) roles.

Although this study provides an important contribution to the debate about the relationship between working memory and updating, there were some limitations. In particular, we used only one measure assessing updating, versus three M capacity tasks. Employment of a more numerous set of updating measures would provide more complete information about its relationship with working memory. Therefore, future studies should replicate our study using other updating tasks in addition to Magic House.

## Figures and Tables

**Table 1:** Descriptive statistics for updating and working memory capacity tasks.

	Min	Max	Mean	S.D.	Skew	Kurtosis
Magic House	4.0	18.0	11.85	3.08	−0.06	−0.55
Mr. Cucumber	0.00	6.00	1.69	0.86	0.57	2.91
Backward Word Span	1.00	5.00	1.94	0.84	0.47	−0.37
Direction Following Task	0.00	3.50	1.76	1.05	−0.09	−0.87

**Table 2:** Correlations between age, updating, and M capacity tasks.

	Magic House	Backward Word Span	Mr. Cucumber	Direction Following Task
Age	0.45 ***	0.68 ***	0.53 ***	0.62 ***
Magic House		0.44 ***	0.38 ***	0.44 ***
Backward Word Span			0.52 ***	0.59 ***
Mr. Cucumber				0.38 ***

Note: *** *p* < .001.

**Table 3:** Cross classification of Magic House performance by M capacity (observed frequencies in boldface; frequencies expected by chance in italics).

	M Capacity (Average of Three Working Memory Tasks)				
		1	2	3	Total
	High	**9** *(23.9)*	**30** *(24.5)*	**21** *(11.6)*	60
**Magic House**	Intermediate	**32** *(30.2)*	**32** *(31.1)*	**12** *(14.7)*	76
	Low	**29** *(15.9)*	**10** *(16.4)*	**1** *(7.7)*	40
	Total	70	72	34	

## Data Availability

The data presented in this study are publicly available in the Open Science Framework at osf.io/9en2z doi:10.17605/OSF.IO/9EN2Z with the following reference: Panesi S. (26 October 2021). On the relation between the development of working memory updating and working memory capacity in preschoolers.

## Notes

1 Research on working memory development keeps flourishing; recently, some relevant studies investigated issues such as verbal coding, rehearsal, and the interference and distraction from irrelevant sounds (e.g., Elliott et al. 2016, 2021; Joseph et al. 2018; Röer et al. 2018). However, these lines of working memory research are not examined in this article, which only focuses on working memory capacity and its relation with updating.

2 Two other meta-analytic studies (Kassai et al. 2019; Sala and Gobet 2017) are potentially relevant here. However, they regard working memory globally as an executive function, without specifying whether the working memory trainings involved updating or capacity tasks. Moreover, Kassai et al. (2019) define far transfer as an effect of working memory training on inhibition or shifting performance—not on intelligence or academic performance. Therefore, the implications of these two studies for our argument are unclear.

3 The 118 children from the Panesi and Morra (2020) study took part in four sessions with various language, drawing, working memory, and executive function tasks. They performed the Direction Following Task in the first session, after three drawing tasks; the Mr. Cucumber and the Backward Word Span tasks were performed in the third session, followed by a language task; and the Magic House task was performed in the fourth session, between a shifting task (the Dimensional Change Card Sort) and a language task (the Test of Grammar Comprehension for Children). The other 58 children took part in four sessions with a large battery of working memory and executive function tasks. The placement of tasks in different sessions and the task order were counterbalanced across participants. This battery included no other working memory capacity measure than the three reported here; it also included three updating tasks, i.e., a Self-Ordered Pointing task and a child version of the Keep Track task, in addition to the Magic House task reported here.

4 To check whether we could safely assume that the relation of each test to age was linear, we performed a series of regression analyses, in which each measure was, in turn, the dependent variable. We inserted age as a predictor (which was highly significant, *p* < .001 for every test) and then, as potential predictors, using a stepwise method, we considered age-squared, age-cube, square root of age, logarithm of age, and a dummy variable (0 for children younger than five years, 1 for at least five years of age). None of these potential predictors entered the regression equation with a significant contribution; the highest *t*-value that we found was *t*(173) = .59, *p* > .55, for the logarithm of age with respect to the Mr. Cucumber task. In sum, there was no evidence for any non-linear component in the relation between any test and age.
